# Can HLA-DRB4 Help to Identify Asthmatic Patients at Risk of Churg-Strauss Syndrome?

**DOI:** 10.1155/2014/843804

**Published:** 2014-03-06

**Authors:** P. Bottero, F. Motta, M. Bonini, F. Vecchio, F. Ierna, I. Cuppari, R. A. Sinico

**Affiliations:** ^1^Allergy and Clinical Immunology Outpatients Clinic, Azienda Ospedaliera Legnano, Ospedale di Magenta, Via Donatore di Sangue 50, Magenta, 20013 Milan, Italy; ^2^Immunohematology Unit, Azienda Ospedaliera Legnano, Ospedale di Magenta, Via Donatore di sangue 50, Magenta, 20013 Milan, Italy; ^3^Hygienics and Public Health Unit, Azienda Sanitaria Locale Provincia Milano1, Via Spagliardi 19, Parabiago, 20014 Milan, Italy; ^4^Clinical Immunology Unit and Renal Unit, Department of Medicine, Azienda Ospedaliera Ospedale San Carlo Borromeo, Via Pio Secondo 3, 20153 Milan, Italy

## Abstract

HLA-DRB4 gene is associated with Churg-Strauss syndrome (CSS), a systemic eosinophilic vasculitis with a prodromal phase characterized by severe asthma, eosinophilia, nasal polyposis, and sinusitis. Aim of this study was to evaluate if the presence of HLA-DRB4 in asthmatic patients is associated with a clinical picture resembling that of the prodromal phase of CSS. HLA-DRB1 was determined in a cohort of 159 asthmatic patients and its frequency was compared with that of 1808 blood donors. HLA-DRB4 presence/absence was correlated with clinical features, including sinusitis, nasal polyposis, eosinophils, antiasthmatic drugs, asthma severity, and pulmonary function tests. HLA-DRB4 gene was associated with severe persistent asthma before treatment (*P* < 0.02), near fatal or severe hypoxemic asthma (*P* < 0.01), sinusitis (*P* < 0.01), nasal polyposis (*P* < 0.01), number of patients with eosinophils >1000/**μ**l: (*P* < 0.05), need of beclomethasone >1000–2000 **μ**g/daily (*P* < 0.001), use of a third controller (*P* < 0.05), and oral prednisone (*P* < 0.02). HLA-DRB4 gene is associated in asthmatic patients with a clinical picture characterized by asthma severity, sinusitis, nasal polyposis, and eosinophilia closely resembling that of the prodromal phase of CSS and might be useful to suspect corticosteroids-masked cases of CSS.

## 1. Introduction

Chug-Strauss syndrome (CSS) is a rare form of eosinophilic necrotising antineutrophil cytoplasmic antibodies (ANCA) associated with vasculitis affecting small to medium sized vessels [1]. In most patients, it is characterized by a prodromal phase, in which severe adult onset asthma is the main feature with rhinosinusitis and nasal polyposis, a second phase, with peripheral blood and tissue eosinophilia, and, finally, the systemic vasculitic phase [1]. In accordance with the American College of Rheumatology (ACR) 1990, asthma, eosinophilia >10%, mononeuropathy or polyneuropathy, pulmonary infiltrates nonfixed, paranasal sinuses abnormality, and biopsy with extravascular eosinophils are the major classification criteria of the syndrome.

The etiology of CSS is not known. However, its pathogenesis is considered multifactorial: the disease can be triggered by exposure to allergens or drugs; Th2 responses are prominent, with upregulation of several cytokines such as IL-4, IL-13, and IL-5; however, Th1 and Th17 responses are not negligible. In particular, Th17 lymphocytes have been involved in the pathogenesis of both autoimmune diseases and allergy/asthma [2] and, interestingly enough, CCS shares features of allergy/asthma and autoimmunity.

A significant prevalence of HLA-DRB4 gene encoding the supertypic HLA-DR53 antigen, which is in strong linkage disequilibrium with the HLA-DRB1∗04, ∗07, and ∗09 alleles, was observed by Vaglio et al. in 48 Italian CSS patients [3], whereas HLA-DRB3 gene, which encodes the HLA-DR52 antigen and is in strong linkage disequilibrium with the HLA-DRB1∗03, ∗11, ∗12, ∗13, and ∗14 alleles, was underrepresented in CSS patients versus controls. Moreover, a higher frequency of HLA-DRB4 positive patients was found in ANCA positive patients [3].

These results were confirmed by Wieczorek et al. [4] in 108 German CSS patients. They also excluded that the HLA-DRB4 association with CSS was due to linkage disequilibrium with the Wegener's granulomatosis-linked HLA-DPB1 locus.

Both Vaglio et al. and Wieczorek et al. compared their results in CSS versus healthy controls but a control group constituted by asthmatic subjects was not included. Since several associations between HL-DR genes and asthma have been reported [5–8], and taking into account that the prevalence of CSS is much higher in adult onset asthma patients than in the general population, the aim of our study was

- to examine the prevalence of HLA-DRB4 in a cohort of asthmatic patients;
- to investigate potential associations of HLA-DRB4 presence with a clinical phenotype resembling that of the prodromal phase of CSS.

## 2. Materials and Methods

This was a retrospective study conducted in a cohort of 159 consecutive Caucasian ANCA negative adult (104 females and 55 males) asthmatic patients from Allergy and Clinical Immunology Outpatient Clinic.

Asthma had been diagnosed and classified in conformity with Global Strategy for Asthma Management and Prevention (GINA) 2007 (update) [9].

Alternative causes of recurrent wheezing had been excluded: exception to this rule was chronic rhinosinusitis and nasal polyposis because of the high prevalence of these conditions in CSS. Chronic rhinosinusitis (with or without nasal polyps) was diagnosed when the symptoms as nasal congestion/obstruction/blockage (or anterior-/posterior-nasal drip) with facial pain/pressure or reduction/loss of smell were lasting for >12 weeks and by nasal endoscopy [10]; computed tomography was performed in patients with severe symptoms or undergoing surgery.

Depending on the asthma severity, the included patients had a six-monthly, quarterly, or more frequently follow-up for not less than two years for a mean follow-up of 4.

Patients not punctually respecting the planned medical examinations and those not believed to be generally drugs complaints had not been included in the study. 

1808 age- and gender-matched white Italian blood donors served as controls (Contr).

The study was conducted in accordance with the declaration of Helsinki and approved by Local Ethical Committee. Informed consent was obtained from the subjects included in the study.

### 2.1. Physiologic Assessment

Pulmonary function tests were performed with standard protocols and following European Respiratory Society guidelines. Values were expressed as a percentage of predicted values, except for forced expiratory volume in 1 s (FEV1)/forced vital capacity (FVC) which was expressed as absolute percentage. All measures of lung volumes were performed in our department. FEV1 and FEV1/FVC were assessed 2 hours after the usual morning treatment; in the patients with FEV1 >80%, the measure was performed again 10–15 min after the administration of short acting inhaled beta-2-agonist. Effect of beta-agonists was expressed as FEV1 after bronchodilator—FEV1 at baseline/FEV1 predicted [11]. Persistent airflow obstruction was defined by a postbronchodilator FEV1/FVC ratio <70% of predicted value with FEV1 <80% of predicted value according to the criteria of the global initiative for chronic obstructive lung disease (GOLD) [12].

### 2.2. HLA Genotyping

Genomic DNA was extracted from EDTA (5 mL) treated peripheral blood samples using the Maxwell 16 Blood DNA Purification System (Promega, Madison, WI, USA) and stored at −20°C until use. Low-resolution genotyping for HLADRB1 loci was performed by polymerase chain reaction (PCR) using sequence-specific primers (One Lambda, Canoga Park, CA, USA). The results were analyzed with Software Fusion 2.0 (One Lambda, Canoga Park, CA, USA). The HLA-DRB4 gene frequency was calculated by adding the frequencies of the HLA-DRB1∗04, ∗07, and ∗09; HLA-DRB1∗03, ∗11, ∗12, ∗13, and ∗14 alleles were added to calculate the HLA-DRB3 gene frequency.

### 2.3. Statistical Analysis

The analyses were performed using MINISTAT statistical software, release 1.1 (Pubblicazioni Medico Scientifiche, Udine, Italy); and only Fisher's exact test was performed using SAS System software (SAS Institute Inc., Cary, NC, USA). The significance of difference between variables was assessed by the Wilcoxon test, for independent samples, for continuously distributed variables and the chi-square (*χ* ^2^) test or Fisher's exact test, when *χ* ^2^ may not be a valid test, for categorical variables. *P* values less than 0.05 were considered significant. Odds ratios (OR) and 95% confidence interval (95% CI) were calculated.

### 2.4. HLA-DRB4 and Clinical Correlations

The medical records of the patients were revised in search of potential associations with HLA- DRB4. The inquired data are reported in Table 1.

## 3. Results and Discussion

### 3.1. Results

#### 3.1.1. HLA-DRB1 Findings

There were no statistically significant differences in HLA-DRB1 frequency between controls and the asthmatic patients; 60 patients were DRB4 positive and 99 DRB4 negative (Table 2).

#### 3.1.2. HLA-DRB4 and Clinical Correlations

There were no statistically significant differences between HLA-DRB4 positive and negative patients in the following items:

- sex (23 males and 37 females versus 32 and 67, resp.; *P* = 0.43);
- age of asthma onset (interquartile 25–75% range; 32.5 (23.71–45.39) versus 31.0 (19.4–43.0); *P* = 0.32);
- asthma onset age > 45 years (21.7% versus 17.2%; *P* = 0.48);
- prevalence of respiratory allergy (75% versus 69.7%; *P* = 0.47);
- years of follow-up (interquartile 25–75% range; 3.88 (2.73–8.44) versus 4.32 (2.67–6.94); *P* = 0.75).

However, 16 of 60 (26.7%) HLA-DRB4 positive patients had been classified as affected by severe persistent asthma before treatment versus 12 of 99 (12.1%) HLA-DRB4 negative patients (*P* < 0.02).

Moreover, HLA-DRB4 positive patients had a higher frequency of

- a history of emergency admission for near fatal or severe hypoxemic asthma: 12 of 60 (20%) versus 5 of 99 (5%) (*P* < 0.01).
- Diagnosis of chronic rhinosinusitis: 21 of 60 (35%) versus 16 of 99 (16.2%) (*P* < 0.01).
- History of nasal polyposis and polypectomy: 19 of 60 (31.7%) versus 14 of 99 (14.1%) (*P* < 0.01).
- Number of patients needing beclomethasone > 1000–2000 *μ*g/daily: 25 of 60 (41.7%) versus 15 of 99 (15%) (*P* < 0.001).
- Number of patients needing a third controller: 13 of 60 (21.7%) versus 10 of 99 (10.1%) (*P* < 0.05).
- Number of patients needing continuous or near continuous oral prednisone: 10 of 60 (16.7%) versus 5 of 99 (5.1%) (*P* < 0.02).

Concerning physiologic assessment, final FEV1 < 80% was present in 9 of 60 HLA-DRB4 positive patients (15%) versus 5 of 99 (5.1%) (*P* < 0.05), whereas the number of patients with persistent airflow obstruction was higher in HLA-DRB4 positive patients (6 of 60 = 10% versus 2 of 99 = 2%) not reaching statistical significance (*P* = 0.06) (Table 3).

There were no statistically significant differences in the medians of eosinophils both as percentage and as absolute number but there was a statistically significant difference in the number of patients with eosinophils >1000/*μ*L (14 of 60 (23.3%) HLA-DRB4 positive patients versus 11 of 99 (11.1%), *P* < 0.05).

The use of long acting inhaled *β*2-agonist (LABA) was not different in the 2 subgroups.

### 3.2. Discussion

The human leukocyte antigens (HLA) are encoded by chromosome 6 and they are involved in presenting peptides to T cells; as a consequence, they could play a role in the development of allergic inflammatory responses. Particularly, HLA-DRB1 genes have been involved in asthma severity in children [5, 6] and susceptibility to occupational asthma [7] and allergic bronchopulmonary aspergillosis [8]. They are implicated also in nasal polyposis and, in particular, the HLA-DRB1∗04 allele has been associated with sinonasal polyposis in a study on the Mexican Mestizo population [13] and in a group of Turkish patients with nasal polyposis, asthma, and aspirin salicylic acid triad [14].

It is of note that the prodromal phase of CSS is characterized by adult onset asthma associated with rhinitis, sinusitis, nasal polyps, and eosinophilia. Asthma is usually severe [15] and many patients require oral corticosteroids to achieve asthma control. In addition, residual asthma after the treatment of the vasculitic phase remains a main problem [1].

Chronic rhinosinusitis and nasal polyposis are very common in CSS [16] and paranasal sinus abnormalities are included in CSS 1990 ACR classification criteria.

Interestingly, in two independent studies from Italy [3] and Germany [4] HLA-DRB4 gene has been significantly associated with CSS whereas HLA-DRB3 gene was significantly underrepresented.

In the Italian study, the presence of HLA-DRB4 was mainly due to the DRB1∗07 allele and the low prevalence of HLA-DRB3 was due to the low presence of DRB1∗13 allele although its significance was lost after correction for multiple comparisons.

In the German study, the presence of HLA-DRB4 was strongly associated with DRB1∗04 allele and HLA-DRB3 was underrepresented mainly for the low presence of DRB1∗13 allele reaching a statistically significant difference. In both studies, a control population of asthmatic patients was not evaluated.

Therefore, the aim of this study was not to face the role of HLA in asthma and even less the complexity of asthma genetics but to evaluate if the presence of HLA-DRB4 might be useful to identify, on clinical grounds, a subpopulation of asthmatic patients at higher risk of developing CSS.

In our study, we could not find any statistically significant difference in HLA-DRB1 allelic frequency between the asthmatic patients and controls but, in our population of asthmatic patients, HLA-DRB4 positivity seems to identify a subgroup of patients strikingly resembling the clinical picture of the prodromal phase of CSS.

Actually, the HLA-DRB4 positive patients are characterized by prevalence of severe persistent asthma before treatment, history of severe hypoxemic or near fatal asthma, diagnosis of chronic rhinosinusitis, history of nasal polyps and, moreover, their need of high doses of inhaled steroids, the use of a third controller (as theophylline) in addition to LABA, and daily oral prednisone to achieve asthma control. However, these results should be considered with caution since, for some variables, there was yield of larger confidence intervals (i.e., history of emergency admission, diagnosis of chronic sinusitis, history of nasal polyposis, patients needing beclomethasone, and patients with continuous prednisone) with less precise estimates of the parameter, probably due to the low number of cases.

Furthermore, the physiologic assessment showed that the number of patients with FEV1 <80 was significantly greater versus HLA-DRB4 negative patients while the number of patients with persistent airflow obstruction, defined by a postbronchodilator FEV1/FVC ratio <70% of predicted value with FEV1 <80% of predicted value, was tendentially greater in HLA-DRB positive patients (10% versus 2%).

The medians of eosinophils were not statistically significantly different but an explanation of that might be given by the greater need of corticosteroids to control asthma in HLA-DRB4 positive patients with a masking effect on eosinophilia; however, in spite of this, the number of patients with eosinophils >1000/*μ*L was significantly higher in HLA-DRB4 positive patients.

Finally, there were no differences in medians of age of asthma onset and in the number of patients with age of asthma onset >45 years.

In the study of Vaglio et al. [3], sinusitis was prevalent in HLA-DRB4 positive patients (64.5%) versus HLA-DRB4 negative patients (41.2%), not reaching a statistically significant difference probably because of the small sample size (48 patients); Wieczorek et al. [4] confirmed the association between CSS and HLA-DRB4 in a larger sample size (102) but they did not reported about clinical associations.

## 4. Conclusions

Our results seem to indicate that the presence of HLA-DRB4 gene in asthmatic patients, even in absence of an evident eosinophilia, could identify a subpopulation in which the risk of developing CSS should be greater than that of the whole cohort. If this hypothesis will be confirmed by long-term follow-up studies, this subgroup of patients should be followed more closely in order to recognize early possible signs/symptoms of CSS. Moreover, recommendations about oral corticosteroids use and tapering as well as about caution in the use of leukotriene modifiers in these patients could be introduced [1].

Finally, ANCA monitoring might be warranted in this subgroup of patients [1].

## Figures and Tables

**Table 1:** The inquired data in the medical records of a cohort of asthmatic patients.

Sex	
Age of asthma onset (median)	
Follow-up (years)	
Diagnosis of respiratory allergy by means of skin prick test or RAST ( radioallergosorbent test)	
History of nasal polyps/nasal polypectomy	
Diagnosis of chronic rhinosinusitis	
Asthma severely persistent before treatment′′	
Emergency admission for severe hypoxemic or near fatal asthma	
Eosinophil number (/µL) (range)^#^	
Eosinophils > 1000/*µ*L^#^	
Eosinophils % (range)^#^	
Beclomethasone > 1000–2000 *μ*g daily^*∧*^	
Long acting inhaled *β*2-agonist (LABA)	
Need of a third controller (as sustained release theophylline)	
Treatment with continuous or near continuous (≥50% of year) prednisone	
Final forced expiratory volume in 1 s (FEV1) expressed as a percentage of predicted value^§^	
Persistent airflow obstruction defined by a postbronchodilator FEV1/FVC ratio < 70% of predicted with FEV1 < 80% of predicted^§^	

′′Defined by daily symptoms, frequent exacerbations, frequent nocturnal asthma symptoms, limitation of physical activities, FEV1 or PEF ≤ 60% predicted, and PEF or FEV1 variability > 30%.

^*∧*^Or equivalents; ^#^maximum value during the follow-up.

^§^is measured 2 hours after usual morning treatment: in case of FEV1 < 80% is measured again after 10–15 min after the administration of short acting inhaled beta-2-agonist.

**Table 2:** Main HLA DRB1 findings in 159 asthmatics patients and 1808 blood donors. Values are the number of positive/2 × number of subjects (allelic frequency %) with the exception of the last two values (gene frequency for patients and controls).

Allele/gene	Patients	Controls	*P*
DRB1∗04	7.5	6.7	0.58
DRB1∗07	12.9	12.1	0.69
DRB1∗09	0.6	0.7	0.89
DRB1∗03	8.8	7.9	0.57
DRB1∗11	30.6	27.4	0.23
DRB1∗12	0.9	1.2	0.69
DRB1∗13	7.9	11.3	0.06
DRB1∗14	6.6	6.3	0.81
DRB4	21.1	19.6	0.52
DRB3	55.6	54.6	0.74
DRB4	37.7	35.4	0.55
DRB3	82.4	80.1	0.48

**Table 3:** Main clinical and laboratory characteristics of the 60 HLA-DRB4 positive and 99 HLA-DRB4 negative asthmatic patients. Categorical variables are reported as percentage.

	DRB4 positive	DRB4 negative	*P*	OR	95% CI
Severe persistent asthma before treatment	26.7	12.1	<0.02	2.64	1.48–6.06
Emergency admission for near fatal or severe hypoxaemic asthma	20.0	5.0	<0.01	4.7	1.57–14.12
Chronic rhinosinusitis	35.0	16.2	<0.01	2.79	1.31–5.93
History of nasal polypectomy	31.7	14.1	<0.01	2.81	1.28–6.17
Patients with eosinophils > 1000/*μ*L	23.3	11.1	<0.05	2.43	1.02–5.79
Beclomethasone > 1000 *μ*g daily°	41.7	15.2	<0.001	4	1.89–8.48
LABA	86.7	76.7	0.12	1.97	0.82–4.74
Third controller	21.7	10.1	<0.05	2.46	1.00–6.04
Continuous or near continuous (>50% of year) prednisone	16.7	5.1	<0.02	3.76	1.22–11.60
Final FEV1 < 80%	15.0	5.0	<0.05	3.32	1.06–10.43
Persistent airflow obstruction	10.0	2.0	0.06	5.94	1.54–30.53

°Or equivalents/daily.
